# Supplementary material for: Range expansion of a fouling species indirectly impacts local species interactions
Source: PeerJ. 2017 Oct 19;5:e3911. doi: 10.7717/peerj.3911 (PMC5651168; doi:10.7717/peerj.3911)

PeerJ Supplemental Material

Cori Speights & Michael McCoy

Data Input.

stone = read.csv(("StoneCrabSponges.csv"), header =T)
stone

## trial scid oysnum sponge hr eaten
## 1 1 1 1 NS 5 1
## 2 1 1 1 NS 24 1
## 3 1 1 1 NS 48 1
## 4 1 2 8 NS 5 0
## 5 1 2 8 NS 24 3
## 6 1 2 8 NS 48 8
## 7 1 3 22 S 5 4
## 8 1 3 22 S 24 16
## 9 1 3 22 S 48 21
## 10 1 4 1 S 5 0
## 11 1 4 1 S 24 0
## 12 1 4 1 S 48 0
## 13 1 5 22 NS 5 5
## 14 1 5 22 NS 24 12
## 15 1 5 22 NS 48 17
## 16 1 6 2 S 5 0
## 17 1 6 2 S 24 1
## 18 1 6 2 S 48 1
## 19 1 7 8 S 5 2
## 20 1 7 8 S 24 2
## 21 1 7 8 S 48 2
## 22 1 8 4 NS 5 3
## 23 1 8 4 NS 24 4
## 24 1 8 4 NS 48 4
## 25 1 9 2 NS 5 0
## 26 1 9 2 NS 24 1
## 27 1 9 2 NS 48 1
## 28 1 10 4 S 5 4
## 29 1 10 4 S 24 4
## 30 1 10 4 S 48 4
## 31 2 1 4 NS 5 4
## 32 2 1 4 NS 24 4
## 33 2 1 4 NS 48 4
## 34 2 2 2 S 5 1
## 35 2 2 2 S 24 2
## 36 2 2 2 S 48 2
## 37 2 3 22 NS 5 3
## 38 2 3 22 NS 24 11
## 39 2 3 22 NS 48 15
## 40 2 4 22 S 5 0
## 41 2 4 22 S 24 0
## 42 2 4 22 S 48 0
## 43 2 5 8 S 5 5
## 44 2 5 8 S 24 7
## 45 2 5 8 S 48 8
## 46 2 6 8 NS 5 2
## 47 2 6 8 NS 24 6
## 48 2 6 8 NS 48 7
## 49 2 7 1 S 5 0
## 50 2 7 1 S 24 0
## 51 2 7 1 S 48 0
## 52 2 8 1 NS 5 1
## 53 2 8 1 NS 24 1
## 54 2 8 1 NS 48 1
## 55 2 9 4 S 5 2
## 56 2 9 4 S 24 4
## 57 2 9 4 S 48 4
## 58 2 10 2 NS 5 2
## 59 2 10 2 NS 24 2
## 60 2 10 2 NS 48 2
## 61 3 1 2 NS 5 2
## 62 3 1 2 NS 24 2
## 63 3 1 2 NS 48 2
## 64 3 2 8 S 5 3
## 65 3 2 8 S 24 8
## 66 3 2 8 S 48 8
## 67 3 3 1 NS 5 1
## 68 3 3 1 NS 24 1
## 69 3 3 1 NS 48 1
## 70 3 4 4 S 5 0
## 71 3 4 4 S 24 2
## 72 3 4 4 S 48 3
## 73 3 5 2 S 5 2
## 74 3 5 2 S 24 2
## 75 3 5 2 S 48 2
## 76 3 6 8 NS 5 1
## 77 3 6 8 NS 24 5
## 78 3 6 8 NS 48 6
## 79 3 7 22 NS 5 3
## 80 3 7 22 NS 24 4
## 81 3 7 22 NS 48 12
## 82 3 8 22 S 5 7
## 83 3 8 22 S 24 16
## 84 3 8 22 S 48 22
## 85 3 9 1 S 5 1
## 86 3 9 1 S 24 1
## 87 3 9 1 S 48 1
## 88 3 10 4 NS 5 4
## 89 3 10 4 NS 24 4
## 90 3 10 4 NS 48 4
## 91 4 1 22 NS 5 3
## 92 4 1 22 NS 24 6
## 93 4 1 22 NS 48 9
## 94 4 2 8 S 5 4
## 95 4 2 8 S 24 7
## 96 4 2 8 S 48 8
## 97 4 3 4 NS 5 4
## 98 4 3 4 NS 24 4
## 99 4 3 4 NS 48 4
## 100 4 4 1 S 5 0
## 101 4 4 1 S 24 1
## 102 4 4 1 S 48 1
## 103 4 5 2 S 5 2
## 104 4 5 2 S 24 2
## 105 4 5 2 S 48 2
## 106 4 6 22 S 5 2
## 107 4 6 22 S 24 8
## 108 4 6 22 S 48 10
## 109 4 7 4 S 5 2
## 110 4 7 4 S 24 4
## 111 4 7 4 S 48 4
## 112 4 8 1 NS 5 1
## 113 4 8 1 NS 24 1
## 114 4 8 1 NS 48 1
## 115 4 9 2 NS 5 1
## 116 4 9 2 NS 24 2
## 117 4 9 2 NS 48 2
## 118 4 10 8 NS 5 7
## 119 4 10 8 NS 24 8
## 120 4 10 8 NS 48 8

Isolating only data taken at the 24 hour mark. After 48 hours crabs at highest treatment (22 oysters) had eaten most/all which would make us unable to use the functional response curves.

new <- droplevels(subset(stone,hr==24))

Necessary Libraries.

library(ggplot2)
library(grid)
library("bbmle")
require("bbmle")
library("emdbook")
require("emdbook")

Rogers random predation model.

rogers.pred <- function(N0,a,h,T) {
 N0 - lambertW(a*h*N0*exp(-a*(T-h*N0)))/(a*h)
}

Necessary variable assignments for the Rogers random predation model.

N0 <- new$oysnum
a <- 1
h <- 0.2
T0 <- 1

eaten <- new$eaten
sdat <- data.frame(N0,eaten,sponge=new$sponge)

Two stone crabs did not eat during experiment or intermediate days so they were removed from the trials and data analysis.

nonew<-new[-c(4,14),]
nosdat<-sdat[-c(4,14),]

Models fit using methods of maximum likelihood using the bbmle package.

(m8 <- mle2(eaten~dbinom(size=N0,prob=rogers.pred(N0,a,h,T)/N0),
 #method="SANN",
 parameters=list(a~sponge, h~sponge),
 start=list(a=c(1.5,1.5),h=c(0.5,0.5)),
 data=c(as.list(nosdat),T=T0)))

(m9 <- mle2(eaten~dbinom(size=N0,prob=rogers.pred(N0,a,h,T)/N0),
 #method="SANN",
 parameters=list(h~sponge),
 start=list(a=c(1.5),h=c(0.5,0.5)),
 data=c(as.list(nosdat),T=T0)))

(m10 <- mle2(eaten~dbinom(size=N0,prob=rogers.pred(N0,a,h,T)/N0),
 #method="SANN",
 parameters=list(a~sponge),
 start=list(a=c(1.5,1.5),h=c(0.5)),
 data=c(as.list(nosdat),T=T0)))

(m11 <- mle2(eaten~dbinom(size=N0,prob=rogers.pred(N0,a,h,T)/N0),
 #method="SANN",
 start=list(a=c(1.5),h=c(0.5)),
 data=c(as.list(nosdat),T=T0)))

Model comparisons using size-corrected Akaike Information Criterion (AICc).

AICctab(m8,m9,m10,m11,delta=T,weights=T,nobs=38)

Calculating the confindence intervals for the attack rate and handling time model parameters.

confint(m8,method="quad")
confint(m9,method="quad")
confint(m10,method="quad")
confint(m11,method="quad")

Calculate mean and standard error for the no sponge treatments.

newns <- droplevels(subset(nonew,sponge=="NS"))
N0 <- newns$oysnum
eaten <- newns$eaten

newns.mean=with(newns,aggregate(eaten,list(c(N0)),mean)) #vector of means
sterr=function(x){sd(x)/sqrt(length(x))} #st. error
newns.err=with(newns,aggregate(eaten,list(c(N0)),sterr)) #vecotr of st. error
xx=data.frame(N0=newns.mean[,1],eaten=newns.mean[,2],err=newns.err[,2]) #data frame
limits=aes(ymax = eaten + err, ymin=eaten - err)

Calculate mean and standard error for the sponge treatments.

news <- droplevels(subset(nonew,sponge=="S"))
N0 <- news$oysnum
eaten <- news$eaten

news.mean=with(news,aggregate(eaten,list(c(N0)),mean)) #vector of means
sterr=function(x){sd(x)/sqrt(length(x))} #st. error
news.err=with(news,aggregate(eaten,list(c(N0)),sterr)) #vecotr of st. error
xy=data.frame(N0=news.mean[,1],eaten=news.mean[,2],err=news.err[,2]) #data frame
limits=aes(ymax = eaten + err, ymin=eaten - err)

Figure code using parameter estimates from the m1 model.

g2=ggplot(xy,aes(N0,eaten))+
 geom_point(col="black",size=9) + ylim(c(0,20))+
 labs(x = "Initial Prey Density",y="Prey Consumed") + geom_errorbar(limits, width=0.25)
g3=g2+ geom_errorbar(limits, width=0.25)+stat_function(fun = function(x,a,h,T) { x - lambertW(a*h*x*exp(-a*(T-h*x)))/(a*h)},
 args=list(a=1.970 ,h=.039,T=1),colour = "black",size=2) +
 theme(panel.grid.minor=element_blank(),panel.grid.major=element_blank())+
 theme(axis.title.x = element_text(face="bold", size=25),
 axis.text.x = element_text( size=20),
 axis.title.y = element_text(face="bold", angle=90,size=25),
 axis.text.y = element_text( size=20))
g4=g3+geom_point(data=xx,aes(y=eaten, x=N0),col="gray",size=9)+geom_errorbar(data=xx,limits, width=0.25)+ stat_function(data=xx,fun = function(x,a,h,T) { x - lambertW(a*h*x*exp(-a*(T-h*x)))/(a*h)},
 args=list(a=4.079 ,h=0.112,T=1),colour = "gray",size=2)

Plot figure.


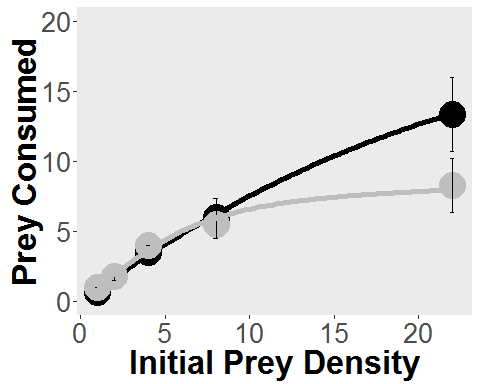

Supplement: Supplemental Information 1 — Data and code used to generate figure and obtain model results. [file peerj-05-3911-s001.docx]
